# Molecular basis and pathways of the Yin-Yang theory in T cell immunity

**DOI:** 10.3389/fimmu.2024.1463399

**Published:** 2024-11-14

**Authors:** Jim Xiang, Scot C. Leary, Zhaojia Wu, Michelle Yu

**Affiliations:** ^1^ Cancer Research Cluster, Saskatchewan Cancer Agency, Saskatoon, SK, Canada; ^2^ Division of Oncology, College of Medicine, University of Saskatchewan, Saskatoon, SK, Canada; ^3^ Department of Biochemistry, Microbiology and Immunology, College of Medicine, University of Saskatchewan, Saskatoon, SK, Canada

**Keywords:** Yin-Yang theory, T cell immunity, molecular mechanisms, signaling pathways, mTORC1

## 1 Introduction

The Yin-Yang theory represents a fundamental principle in traditional Chinese medicine (TCM), guiding its diagnosis and treatment of diseases (1). In the latter context, the Yin element refers to reserve, healing, resting or quiescence, passive or inhibitory factors and negative regulation while the Yang element speaks to consumption, working or activeness, growth or promoting factors and positive regulation. The theory posits that a balance between these two opposing forces becomes the premise of a healthy body (1). The “Qi”, a special energy flowing in the body, is a driver of the defense system in TCM (1), and is analogous to the protective immune system in Western medicine (WM). However, there is a huge gap between the conceptional connection of TCM and WM.

The Yin-Yang theory has been applied historically to interpret immune system biology mostly from the perspective of the counteractive features of these two immune elements or responses. For example, the suppressive CTLA-4 and immunogenic CD28 have been described as the Yin and Yang of T cell co-stimulation (2). Moreover, CD4^+^ Treg and CD4^+^ Th1 cells have been considered to belong to Yin and Yang CD4^+^ T cell subsets, where the former and latter cells contribute to tolerant and active immune responses, respectively (3). More recently, the regulators AMPKα1 and mTORC1 have been proposed to represent Yin and Yang energy sensors, with mTORC1 signaling the availability of nutrients and promoting immune stimuli that support cell proliferation through glycolysis and AMPKα1 signaling a lack of nutrients and inhibiting cell growth through FAO metabolism (4). However, the molecular basis and pathways underpinning the Yin-Yang theory in T cell immunity remain incompletely understood.

**Figure 1:**
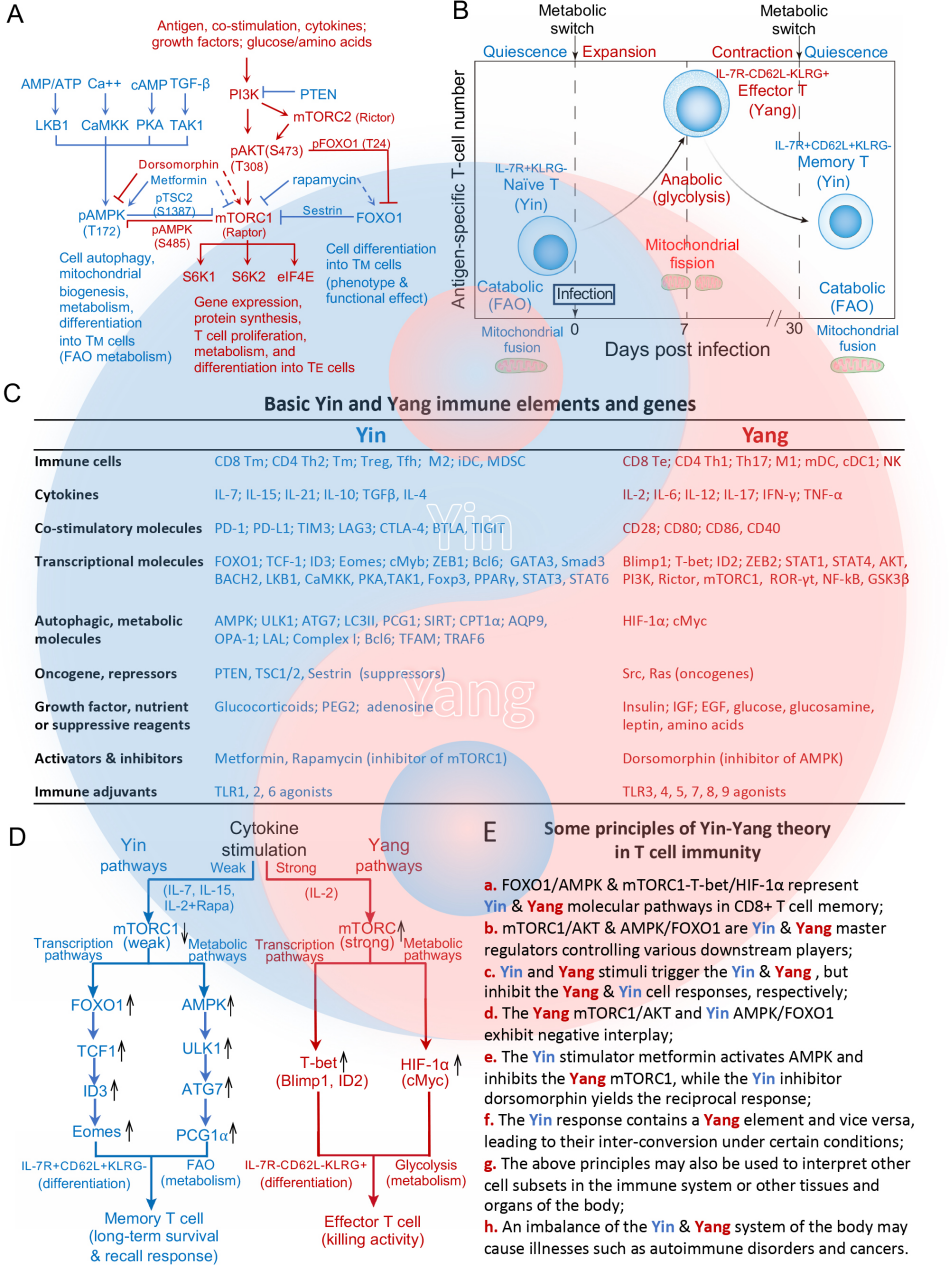
Molecular basis and pathways of the Yin-Yang theory in T cell differentiation and metabolism. **(A)** Schematic diagram illustrating the major, upstream molecular pathways that regulate the activity of the master regulators Yang AKT and mTORC1 and Yin AMPK and FOXO1, which collectively control CD8^+^ T cell immunity. Three immune signals, growth factors or nutrients trigger the Yang molecular PI3K-AKT-mTORC1 pathway to control gene expression, protein systhesis and CD8^+^ T_E_ cell differentiation. AMPK modulates the necessary changes in autophagic flux and FAO metabolism to support CD8^+^ T_M_ cell differentiation. Yin FOXO1 works in concert with Yin AMPK to regulate development of the CD8^+^ T_M_ cell phenotype and desired functional effect. Yin AMPKα1/FOXO1 and Yang AKT/mTORC1 exhibit a negative interaction. The Yin AMPK activator metformin activates AMPK (solid line), but inhibits the Yang mTORC1 (dotted line) while the Yang mTORC1 inhibitor rapamycin inhibits mTORC1 (solid line), but activates the Yin FOXO1 (dotted line). **(B)** Metabolic transitions of CD8^+^ T cells at different stages of the immune response. Post-infection, quiescent naïve CD8^+^ T cells enter a developmental program including T cell expansion followed by a contraction phase. During this latter phase, the majority of IL-7R^-^CD62L^-^KLRG^+^ T_E_ cells die of apoptosis while the remaining T cells differentiate into long-lived IL-7R^+^CD62L^+^KLRG^-^ T_M_ cells. To meet the bioenergetic demand of T_E_ cells during T cell expansion and contration phases, naïve T cells switch from mitochondrial respiration with mitochondrial fusion to glycolysis of T_E_ cells with mitochondrial fission. During the memory phase, the metabolic program reverts to catabolic FAO as T_E_ cells gradually transition to T_M_ cells with mitochondrial fusion. **(C)** Basic Yin and Yang immune elements (cells, cytokines, costimulations, growth factors, nutrients, suppressive reagents, activators or inhibitors) and genes in T cell immunity. **(D)** Schematic diagram of how strong or weak strengths of mTORC1 signaling give rise to distinct T cell subtypes. The Yang IL-2 cytokine elicits strong mTORC1 signaling to promote the differentiation of naïve T cells into glycolytic Yang IL-7^-^CD62L^-^KLRG^+^ T_E_ cells by activating the Yang transcriptional T-bet and Yang metabolic HIF-1α pathways. In contrast, the Yin IL-7/IL and 15/IL-2+Rapa cytokines promote the differentiation of naïve T cells into Yin T_M_ cells that rely on FAO metabolism *via* activation of the Yin transcriptional FOXO1-TCF1-ID3-Eomes and Yin metabolic AMPK-ULK1-ATG7-PCG1α pathways. **(E)** Some basic principles of the Yin-Yang theory in T cell immunity are summarized. All graphs, diagrams and characters in blue and red color represent Yin and Yang, respectively.

mTORC1 is an evolutionarily conserved serine/threonine kinase whose ability to sense three major immune signals (i.e. antigen, co-stimulation, and cytokines) and a variety of environmental cues (e.g. growth factors and nutrient status) allows it to act as a master regulator. Its activity is modulated in a phosphorylation-dependent manner by PI3K-Akt signaling. PI3K phosphorylates PIP2 at the cell surface to generate PIP3, which recruit and phosphorylate Akt at T_308_ through PDK1 (**Figure 1A**) (5). mTORC2 can also be activated by PI3K through PIP3 and phosphorylated at S_473_ (**Figure 1A**), that confers for a full activation and substrate specificity of Akt (6). The phosphorylation of Akt at T_308_ triggers mTORC1 signaling, which in turn up-regulates the downstream substrates ribosomal S6K1 and S6K2 and eIF4E to modulate protein synthesis, cell proliferation, metabolism and differentiation (**Figure 1A**) (7), whereas the phosphorylation of Akt at S_473_ represses FOXO1 activity through phosphorylation of FOXO1 at T_24_ (**Figure 1A**) (8). Autophagy is a self-recycling process in which cellular constituents are degraded by lysosomes to provide essential anabolic building blocks in support of metabolism and homeostasis under stress conditions (9). Flux through autophagy as well as most branches of intermediary metabolism, including mitochondrial FAO, is regulated by the activity of the evolutionarily conserved energy sensor AMPKα1, which is in turn controlled by LKB1, CaMKK2, PKA and TAK-1 (**Figure 1A**) (9). As such, AMPKα1 is central to CD8^+^ T_M_ cell formation (**Figure 1A**) (9). FOXO1 is another well known, key regulator that controls various aspects of cell development (10) and also contributes to CD8^+^ T_M_ cell differentiation (**Figure 1A**) (8). Both AMPKα1 and FOXO1 were also described as tumor suppressors because of their inhibitory effect on tumor growth (8, 9). Therefore, AMPKα1/FOXO1 and AKT/mTORC1 can be characterized as Yin and Yang master regulators in T cell immunity as well as cell biology (**Figure 1A**).

## 2 Two basic cellular characteristics determine the Yin and Yang immune cell subsets

In modern T-cell biology, two basic features have been well studied; (i) cellular differentiation into a defined phenotype with respect to form and function, and (ii) metabolic fuel preference to provide the energy necessary to support function and sustain homeostasis. These features or characteristics are thus used as a standard manner of defining Yin and Yang cell subsets in immunity and may even be extended conceptually to various tissues or organs of the body. CD8^+^ T-cells play a critical role in immune responses against tumors and infectious diseases. During infection, pathogen-engaged DCs use three distinct signals (antigen, co-stimulation and cytokines) to stimulate catabolic CD8^+^ Tn cells to grow and differentiate into one of two CD8^+^ T-cell subsets; anabolic IL-7R^−^CD62L^−^KLRG1^+^ T_E_ cells which rely on glycolysis, grow rapidly and exhibit effective killing activity, or catabolic and quiescent IL-7R^+^CD62L^+^KLRG1^−^ T_M_ cells which use mild FAO for economical energy supply (**Figure 1B**). Based on the theory, CD8^+^ T_M_ and T_E_ cells can thus be characterized as typical Yin and Yang CD8^+^ T-cells, respectively (**Figures 1A–C**).

## 3 Distinct transcriptional and metabolic pathways control Yin and Yang immune cell differentiation and metabolism

The pro-inflammatory IL-2 and pro-survival IL-7 cytokines (**Figure 1C**) belong to common γ-chain cytokine family. While both trigger the same PI3K-AKT-mTORC1 pathway, IL-2 induces T_E_ cell differentiation and IL-7 promotes T_M_ cell formation (11). We recently sought to elucidate the underlying molecular mechanism, and in so doing demonstrated that IL-2 stimulates strong mTORC1 signaling (Yang IL-2/mTORC1^strong^) due to the persistent expression of cell surface IL-2R, which results in IL-2/T_E_ cell formation *via* activation of the Yang transcriptional mTORC1-T-bet and metabolic mTORC1-HIF-1α pathways (**Figure 1D**) (12). In contrast, we observed that IL-7 stimulates weak mTORC1 signaling (Yin IL-7/mTORC1^weak^) due to the transient expression of cell surface IL-7R, which leads to IL-7/T_M_ cell differentiation *via* activation of the Yin transcriptional FOXO1-TCF1-ID3-Eomes and metabolic AMPKα1-ULK1-ATG7-PCG1α pathways (**Figure 1D**) (12). IL-7/mTORC1^weak^ signaling also up-regulates the expression of AQP9, CPT1α, Complex I, Bcl6, LAL and TFAM, which are some of the metabolic factors necessary for increased reliance on FAO (**Figure 1C**) (12). This emerging signaling model is further supported by our other recent findings that pro-survival IL-15-stimulated Yin IL-15/T_M_ and mTORC1 inhibitor Rapamycin-treated Yin IL-2 (IL-2+Rapa)/T_M_ cells also rely on Yin mTORC1^Weak^ signaling and FAO while exhibiting a T_M_ cell phenotype and long-term survival after their adoptive transfer into C57BL/6 mice (**Figure 1D**) (13–15). Like IL-7, IL-15 similarly promotes CD8^+^ T_M_ cell differentiation by stimulating weak mTORC1 signaling (Yin IL-15/mTORC1^weak^) (10) and activating the coupled Yin transcriptional FOXO1-ID3-Eomes and metabolic AMPKα1-ULK1-ATG7-PCG1α pathways. Importantly, these IL-15/T_M_ cells also showed a robust recall response upon antigen challenge (**Figure 1D**) (13, 16). Our data collectively provide the first evidence that distinct strengths of mTORC1 signaling are able to promote T_E_ and T_M_ cell formation *via* the activation of distinct transcriptional and metabolic pathways (12–15), and shed light on the molecular mechanisms underpinning the Yin-Yang theory in T-cell immunity (**Figures 1D, E**a).

## 4 Some other basic principles of the Yin-Yang relationship relevant to T-cell immunity

Some other, basic principles of the Yin-Yang relationship as it pertains to T cell immunity can also be recognized. For example, the Yang master regulator mTORC1 controls the activity of various transcriptional (T-bet, Blimp-1 and ID2) and metabolic (HIF-1α and cMyc) factors. The Yin master regulators FOXO1 (16) and AMPKα1 similarly exert control over various transcriptional (TCF1, ID3 and Eomes) and metabolic (SIRT1, PGC1α, CPT1α, AQP9, OPA-1, LAL, Complex-I, Bcl6 and TFAM) factors (**Figure 1C**) (8, 12–15). These data indicate that mTORC1 alone or FOXO1 and AMPKα1 in concert act as master regulators of various downstream targets crucial to the transcriptional and metabolic programs required for T_E_ and T_M_ cell formation (**Figure 1E**b).

Various Yin and Yang stimuli including cytokines, co-stimulatory molecules, transcriptional and metabolic regulators, repressors, oncogenes and adjuvants among others trigger the maturation of various immune cell subsets and lead to immunotolerant and immunogenic responses, respectively (**Figures 1C, E**c). For example, the Yin cytokines IL-10 and TGF-β promote the differentiation of CD4^+^ Treg, T_M_ and CD8^+^ T_M_ or M2 cells while the Yang cytokines IL-2, IFN-γ and IL-12 stimulate immunogenic CD4^+^ Th1 and CD8^+^ T_E_ cell responses (**Figure 1C**) (17–20). The Yin co-stimulatory molecules PD-1, CTLA-4, LAG3, TIM3, BTAL and TIGIT or agonists TLR1, 2, 6 promote suppressive immune responses, while the Yang molecules CD28, CD40, CD80 and CD86 or agonists TLR3, 4, 5, 7, 8 and 9 stimulate active immune responses (**Figure 1C**) (21, 22).

The theory argues for negative interplay between the two elements. For example, the Yang regulators mTORC1 and AKT interact with the Yin regulators AMPKα1 and FOXO1 *via* negative feedback loops (10). Our recent data showed that mTORC1^Strong^ signaling inhibits Yin AMPKα1 and FOXO1 expression in IL-2/T_E_ cells, while mTORC1^Weak^-induced expression of AMPKα1 and FOXO1 promotes T cell memory in IL-15/T_M_ cells (13). mTORC1-induced phosphorylation of AMPKα1 at S_485_ blocks its phosphorylation at T_172_ in IL-2/T_E_ cells, whereas AMPKα1-mediated phosphorylation of its downstream target TSC2 at S_1387_ weakens mTORC1 signaling in IL-15/T_M_ cells (**Figure 1A**) (13, 23). In addition, mTORC2-dependent phosphorylation of AKT at S_473_ phosphorylates FOXO1 at T_24_, which inhibits FOXO1 by reducing its nuclear localization (**Figure 1A**) (24). FOXO1 in turn is able to down-regulate mTORC1 signaling *via* its activation of sestrin, a suppressor of mTORC1 (**Figure 1A**) (25). Collectively, these reports confirm the negative interplay between the master regulators Yin FOXO1 or AMPKα1 and Yang AKT/mTORC1 (**Figures 1A, E**d). In support of this principle, the AMPK activator metformin inhibits mTORC1 (26), while the mTORC1 inhibitor Rapa promotes AMPKα1 and FOXO1 activity (**Figures 1A, E**e) (7, 14).

The theory also advances that the Yin response contains some Yang elements and *vice versa*, which facilitates their interconversion under certain conditions (**Figure 1E**f). For example, a basic feature of T_M_ cells is their ability to rapidly proliferate and switch to functional T_E_ cells in a recall response (**Figure 1B**). This interconversion is facilitated by the maintenance of an AKT-dependent, “imprinted” glycolytic potential (27) or a demethylated, epigenetic mark at the IFN-γ promotor (8, 28) inherited from the Yang CD8^+^ T_E_ precursor, either of which leads to an efficient immediate-early recall response of Yin CD8^+^ T_M_ cells upon cognate antigen re-encounter.

Differentiation of distinct immune cell subsets is accompanied by complementary changes in fuel preference. To date, the Yin and Yang metabolic pathways in all immune cell subsets are regulated by the Yin AMPKα1 for FAO and the Yang HIF-1α for glycolytic metabolism. In contrast, the Yin and Yang transcriptional factors controlling differentiation into a defined form and function may differ across immune cell subsets. For example, Yin GATA3 is stimulated in CD4^+^ Th2 which use FAO while Yang T-bet is triggered in CD4^+^ Th1 cells which rely on glycolysis (17, 29). Similarly, Yin Foxp3, Smad3 and FOXO1 are activated in FAO reliant CD4^+^ Treg cells while Yang ROR-γt is stimulated in glycolytic CD4^+^ Th17 cells (**Figure 1C**) (17, 30). Finally, Yin STAT6 and PPAR-γ are up-regulated in FAO utilizing tolerant M2 cells while Yang STAT1 and NF-κB are stimulated in immunogenic M1 cells relying on glycolysis (**Figure 1C**) (18). These data illustrate that the above principle can be used to interpret various cell subsets of the immune system and may perhaps be extended to cell subsets in various tissues and organs of the body (**Figure 1E**g).

The dynamic balance of Yin and Yang keeps the body healthy, and illness may result when there is an upset within or between these two systems (1, 31, 32). A deficiency in CD4^+^ Treg cells or inhibitory co-stimulation and a more active Yang T_E_ cell response derived from some strong vaccinations can induce detrimental autoimmune and lymphoproliferative diseases, respectively (33–35), while deficiencies in T-cell immunity may underlie infectious and cancerous diseases (**Figure 1E**h) (36).

## 5 Conclusion

Our current knowledge of various immune elements and genes (**Figure 1C**) emphasizes the importance of the interplay between Yin and Yang molecular pathways that control immune responses or underlie various immune disorders (31, 32). Yet additional, integrative studies of large datasets using artificial intelligence or other technological approaches to analyze and interpret various genes and cell subsets are required to further improve our understanding of the molecular bases and pathways of the Yin-Yang theory in T-cell immunity in the context of human health and disease (37, 38). Nevertheless, this nascent model provides a new and simple guide with which to categorize genes into Yin and Yang groups, to facilitate the analysis of complex genetic networks and interactions between genetic elements and molecular pathways. Therefore, this model may act as a novel platform for drug innovation to combat various diseases (37, 38). Future studies in this direction should further strengthen the conceptional connection between TCM and WM, that will be greatly beneficial to human health.

## Glossary

AMPKα1: adenosine monophosphate-activated protein kinase-α1
AQP9: aquaporin-9
ATG7: autophagy-related gene-7
BTLA: B and T lymphocyte attenuator
CaMKK2: Ca^++^/calmodulin-activated protein kinase kinase-2
cDC1: conventional type-1 dendritic cell
CPT1α: carnitine palmitoyl transferase-1α
CTLA-4: cytotoxic T lymphocyte-associated protein-4
DC: dendritic cell
eIF4E: eukaryotic translation initiation factor-4E
FAO: fatty acid oxidation
FOXO1: forkhead box-O1
Foxp3: forkhead box-P3
iDC: immature dendritic cell
GATA3: GATA-binding protein-3
GSK3β: glycogen synthase kinase-3 beta
ID3: DNA binding-3
KLRG1: killer cell lectin-like receptor G1
LAG3: lymphocyte-activation gene-3
LAL: lysosomal acid lipase
LKB1: liver kinase-B1
M1: type-1 macrophage
M2: type-2 macrophage
mDC: mature dendritic cell
mTORC1: mammalian target of rapamycin complex-1
NF-κB1: nuclear-factor kappa-B subunit-1
OPA-1: optic atrophy-1
PCG1α: peroxisome proliferator-activated receptor-γ coactivator-1α
PD-1: programmed cell death protein-1
PDK1: phosphoinositide-dependent kinase-1
PKA: cAMP-activated protein kinase A
PI3K: PI3 kinase
PIP2: phosphatidylinositol 4,5-bisphosphate
PIP3: phosphatidylinositol 3,4,5-trisphosphate
PPAR-γ: peroxisome proliferator-activated receptor-γ
PTEN: phosphatase and tension homolog
Rapa: rapamycin
S6K1: S6 kinase-1
S6K2: S6 kinase-2
SIRT1: silent information-regulator of transcription-1
STAT6: signal transducer/transcription activator-6
TAK-1: TGF-β-activated kinase-1
TCF1: T cell factor-1
T_E_: effector T
TFAM: mitochondrial transcription factor-A
Th1: type-1 helper T
Th2: type-2 helper T
TIGIT: T cell immunoglobulin and ITIM domain
TIM3: timeless-3
TLR: Toll-like receptor
T_M_: memory T
Tn: naïve T
Treg: regulatory T
TSC2: TSC complex subunit 2
ROR-γt: retinoid-related orphan receptor-γt
ULK1: Unc-51-like autophagy-activating kinase-1.
